# Pregnancy Complications Associated With Bacterial Vaginosis and Their Estimated Costs

**DOI:** 10.1155/S1064744995000500

**Published:** 1995

**Authors:** MerriKay A. Oleen-Burkey, Sharon L. Hillier

**Affiliations:** 1 Worldwide Health Care Economic and Policy Research The Upjohn Company Kalamazoo MI 49001 USA; 2 Department of Obstetrics, Gynecology, and Reproductive Sciences University of Pittsburgh/Magee-Womens Hospital Pittsburgh PA USA

## Abstract

*Objective:* This study was undertaken to estimate the annual direct costs of complications associated with bacterial vaginosis (BV) in pregnant women in the United States.

*Methods:* An economic model was developed from evidence in the published literature linking perinatal complications to BV. The estimates of attributable risks were applied to the estimated population of pregnant women in the United States in 1993. The charge data from a database of hospital utilization information were then used to estimate the direct costs of each pregnancy complication and the total direct costs associated with BV.

*Results:* Under the assumptions of our model, the direct costs of preterm labor, preterm delivery, the attendant low birth weight (LBW), and other perinatal complications associated with BV were estimated at nearly $1.0 billion in 1993. Over 40% of the total cost was associated with preterm delivery and intensive care of LBW infants, while another 24.5% of the cost was related to preterm labor.

*Conclusions:* If the current frequency of BV among pregnant women persists and BV is not detected and treated during pregnancy, the projected annual costs will reach $1.4 billion by the year 2000. Reducing the heavy economic burden associated with BV in pregnant women will require the establishment of effective screening and treatment regimens.


Infectious Diseases in Obstetrics and Gynecology 3:149–157 (1995)
© 1995 Wiley-Liss, Inc.

### Pregnancy Complications Associated With Bacterial Vaginosis and Their Estimated Costs

**MerriKay A. Oleen-Burkey and Sharon L. Hillier**

*Worldwide Health Care Economic and Policy Research, The Upjohn Company, Kalamazoo, MI (M.A.O.-B.), and Department of Obstetrics, Gynecology, and Reproductive Sciences, University of Pittsburgh/Magee-Womens Hospital, Pittsburgh, PA (S.L.H.)*

### Abstract

***Objective:*** This study was undertaken to estimate the annual direct costs of complications associated with bacterial vaginosis (BV) in pregnant women in the United States.

***Methods:*** An economic model was developed from evidence in the published literature linking perinatal complications to BV. The estimates of attributable risks were applied to the estimated population of pregnant women in the United States in 1993. The charge data from a database of hospital utilization information were then used to estimate the direct costs of each pregnancy complication and the total direct costs associated with BV.

***Results:*** Under the assumptions of our model, the direct costs of preterm labor, preterm delivery, the attendant low birth weight (LBW), and other perinatal complications associated with BV were estimated at nearly $1.0 billion in 1993. Over 40% of the total cost was associated with preterm delivery and intensive care of LBW infants, while another 24.5% of the cost was related to preterm labor.

***Conclusions:*** If the current frequency of BV among pregnant women persists and BV is not detected and treated during pregnancy, the projected annual costs will reach $1.4 billion by the year 2000. Reducing the heavy economic burden associated with BV in pregnant women will require the establishment of effective screening and treatment regimens. © 1995 Wiley-Liss, Inc.

Key words

Preterm labor, preterm delivery, attributable risk, direct costs

Over the past decade, a growing body of evidence has suggested that bacterial vaginosis (BV) increases the risk of preterm birth and other perinatal complications. To date, 6 published studies have reported an increased risk of preterm birth among women with this common vaginal syndrome. The link between preterm birth and BV reported in these case-control[1,2] or cohort[3–6] studies supports a strong epidemiologic consistency. These studies were conducted in populations of pregnant women in the United States, Scandinavia, the United Kingdom, and Indonesia, representing all ethnic groups and socioeconomic strata. While the risks of preterm birth have ranged from 2.0 to 6.9 in these studies,[1–6] the link between preterm birth and BV found in these studies cannot be accounted for by other risk factors such as smoking, history of preterm birth, or access to prenatal care

Address correspondence/reprint requests to Dr. MerriKay A. Oleen-Burkey, The Upjohn Company, 9352-298-318, Kalamazoo, MI 49001.

This project was funded by The Upjohn Company, Kalamazoo, MI. Basic data for use in this study were supplied by HCIA, Inc., Ann Arbor, MI. These data were supplied only at the request and upon the authorization of the hospitals whose data were used. Any analysis, interpretation, or conclusion based on these data is solely that of Worldwide Health Care Economic and Policy Research, The Upjohn Company. HCIA, Inc. specifically disclaims responsibility for any such analysis, interpretation, or conclusion.

Received June 20, 1995
Accepted October 2, 1995

because a similar link between BV and prematurity has been established in women without these risk factors for preterm birth.

The mechanism by which BV causes preterm delivery or low birth weight (LBW) is not known. However, a substantial body of data demonstrates a link between BV and upper-genital-tract infection in pregnancy, including histologic chorioamnionitis,[7] infection of the chorioamnion,[7] and amniotic-fluid infection.[5,8,9] These infections are common complications of preterm delivery and predictors of higher neonatal morbidity. BV has also been linked with an increased risk of postcesarean endometritis.[9]

If BV or other infectious morbidity represents a preventable cause of preterm birth, the screening and treatment of women with this syndrome would prevent the health care costs associated with these perinatal complications. The present study was undertaken to estimate the costs associated with preterm birth and other infectious complications of BV in the absence of screening and treatment options.

In this report, we present an estimate of the annual direct economic costs of BV in pregnant women in the United States based on an analysis of the published research and a database of hospital discharge records jointly maintained by the Commission on Professional and Hospital Activities (CPHA) and Healthcare Knowledge Resources (HCIA, Inc., Ann Arbor, MI). For this study, we have assumed an etiologic role for BV in pregnancy complications. The direct costs in our analysis refer to health care costs for the goods and services used to treat the putative effects of BV. The charges for medical care are used to approximate these costs.

### SUBJECTS AND METHODS

### Frequency of BV in Pregnant Women

The population projections from the U.S. Bureau of the Census provide an estimate of the number of U.S. women of childbearing age (15–44 years old) in 1993 of 58.8 million[10] The U.S. fertility rate of 67 live births/1,000 women (15–44 years old) has remained relatively stable in recent years. Assuming that all live births are singletons, we can estimate that 3.9 million pregnant women had live births in the United States in 1993. Eschenbach[11] estimates that 15–20% of pregnant women have BV. Using the low end of this range, we can estimate conservatively that there were 585,000 cases of BV in the pregnant population in 1993.

### Frequency of Specific Perinatal Complications and Risk Attributable to BV

The costs of BV-associated infections in pregnant women can be attributed to episodes of preterm labor and delivery with or without premature rupture of the membranes (PROM) and the associated delivery of LBW infants. The link between BV and LBW was assumed to be due to preterm LBW since BV was not linked to intrauterine growth retardation in a cohort of over 13,000 women.[12] In addition, BV in pregnant women is associated with amniotic-fluid infection and postpartum endometritis and their attendant costs. Table 1 summarizes the results of our literature search for the strength of association between BV and each of these perinatal complications.[1–6,8,9,13,14] The annual frequency of the diagnosis of these perinatal complications in the U.S. pregnant population and the proportion of each complication that might be attributed to BV are presented in Table 2.[1–6,8,9,14,15]

The risk of a complication attributable to BV was not consistently available in the literature we included in our investigation. Using the available data on the incidence of a complication in the study population and in those without BV, we calculated an attributable risk. If more than one attributable risk was available for a complication, a U.S. population attributable risk specific to BV was given the highest priority. If several attributable risks could be calculated for a complication from individual studies, a weighted average was used.

### Direct Costs

The cost estimates for the complications of pregnancy related to BV were supplied by HCIA, Inc., which provides database services utilizing the clinical database of CPHA. CPHA's clinical database is nationally recognized as a major source for comprehensive diagnosis and procedure data and clinical utilization norms and standards. Approximately 1,000 short-term, nonfederal, general hospitals in the United States submit data on a regular basis to CPHA.

All hospitals submitting information with valid charge data during the calendar year 1991 were eligible for this study. The actual occurrences of specific labor, delivery, and newborn diagnostic

TABLE 1. Evidence linking perinatal complications to BV

| Reference | No. | Year | OR or RR (95% CI)[a] | P value |
|---|---|---|---|---|
| Preterm labor | | | | |
| Gravett et al.[3] | 534 | 1986 | OR = 2.0 (1.1–3.5) | <0.05 |
| McGregor et al.[14] | 202 | 1990 | RR = 2.6 (1.1–6.5) | 0.03 |
| Kurki et al.[4] | 790 | 1992 | OR = 2.6 (1.3–4.9) | <0.01 |
| Preterm delivery | | | | |
| Gravett et al.[13] | 88 | 1986 | OR = 3.8 (1.2–11.6) | <0.05 |
| Martius et al.[1] | 212 | 1988 | OR = 2.3 (1.1–5.0) | 0.03 |
| Kurki et al.[4] | 790 | 1992 | OR = 6.9 (2.5–18.8) | <0.001 |
| Riduan et al.[5] | 490 | 1993 | OR = 2.0 (1.0–3.9) | <0.05 |
| Hay et al.[6] | 467 | 1994 | OR = 5.5 (2.3–13.3) | <0.01 |
| Holst et al.[2] | 87 | 1994 | OR = 2.1 (1.2–3.7) | <0.05 |
| Preterm PROM | | | | |
| Gravett et al.[3] | 534 | 1986 | OR = 2.0 (1.1–3.7) | <0.05 |
| Kurki et al.[4] | 790 | 1992 | OR = 7.3 (1.8–29.4) | <0.001 |
| Amniotic-fluid infection | | | | |
| Gravett et al.[3] | 534 | 1986 | OR = 2.1 (1.2–3.9) | <0.05 |
| Silver et al.[8] | 125 | 1989 | OR = 2.6 (1.1–4.1) | 0.05 |
| Watts et al.[9] | 462 | 1990 | OR = 7.7 (3.4–16.9) | <0.001 |
| Postpartum endometritis | | | | |
| Watts et al.[9] | 462 | 1990 | OR = 5.8 (3.0–10.9) | <0.01 |

[a]OR = odds ratio; RR = relative risk; CI = confidence interval.

TABLE 2. Complications associated with BV in pregnancy

| Complications | Rate of complication (%) | Population attributable risk percent for BV |
|---|---|---|
| Preterm labor[3,4,14] | 10.0 | 23.0 |
| Preterm delivery[1,2,4–6,14] | 8.0 | 30.0 |
| Preterm PROM[3,15] | 2.0 | 19.0 |
| Amniotic-fluid infection[3,8,9] | 6.0 | 15.0 |
| Endometritis[9] | 5.0 | 33.0 |

codes were tallied, along with the mean and standard deviation for the aggregated length of stay and total charges for each principal code. The costs were based on the hospital charges for an admission with each principal diagnostic code, including delivery inpatient charges and pediatric inpatient charges. Contributing data to the report were 407 hospitals representing all sections of the United States.

Based on the 1991 data from HCIA, Inc., the mean estimated charge for threatened premature labor without delivery (ICD-9-CM Code 644.0) was $2,517. These premature labor cases were serious enough to require at least 1 full day of hospitalization, and the mean length of stay was 2.7 days. The mean charge for a normal delivery (ICD-9-CM Code 650) was $2,298, with a mean length of stay of 1.8 days.

The data for complicated deliveries were partitioned into those delivered by cesarean and those delivered vaginally. Within each delivery category, the occurrence of a complication, length of stay, and charge data were presented by the ICD-9-CM code for each complication (preterm delivery, amniotic infection, and major puerperal infection). Since the charges for complications associated with deliveries were incorporated with the delivery charges, we subtracted the mean 1991 charge for a normal delivery from the charges for complicated vaginal deliveries and the mean charge for an uncomplicated cesarean delivery from charges for complicated cesarean deliveries. In order to reflect the proportion of complicated deliveries requiring cesarean (20%) compared with vaginal, we used a weighted average of the charges for vaginal deliveries (80%) and cesarean deliveries (20%) for each complication.

For example, the mean charge for an early onset vaginal delivery (ICD-9-CM Code 644.2), defined as a birth before the completion of 37 weeks gestation, was $3,515, while the mean charge for an early onset cesarean delivery was $8,127. Subtracting the mean charges for uncomplicated deliveries, the mean excess charge for early onset vaginal delivery was $1,217 and the mean excess charge for early onset cesarean delivery was $3,761. The weighted average charge for a preterm delivery, regardless of delivery method, was then estimated to be $1,726 in 1991. A similar method was used to calculate the weighted average charges for early onset of delivery with PROM (ICD-9-CM Code 644.2 + 658.1 or 658.2) which was $2,210 and amniotic-fluid infection (ICD-9-CM Code 658.4) which was $1,888.

Major puerperal infections (ICD-9-CM Code 670.0) include postpartum endometritis which occurs much more frequently in patients undergoing cesarean delivery than in patients who deliver vaginally. As a result, we used a weighted average of charges that was partitioned at 55% for vaginal deliveries and 45% for cesarean deliveries. The estimated charge for postpartum endometritis, regardless of delivery method, was calculated to be $3,081 for 1991.

Since we assumed that BV is associated with the delivery of preterm LBW infants, we incorporated the charge for ICD-9-CM Code 765.1 (other preterm infants) of $19,757 into our cost accounting which represented a mean length of stay for the preterm infant of 18 days. This ICD-9-CM code for preterm LBW represented 95% of the occurrences of LBW in this sample of the database. To estimate the cost of the complication of preterm LBW among these infants, we subtracted the charges for normal and healthy infants. In 1982, Fuchs and Perreault[16] estimated the direct money costs of normal infant medical care as $64 for pediatric care and an average hospital charge of $100 per day or $340 total. Adjusting these costs for medical care inflation over the 10 years and the reduction in length of stay from a mean of 3.4 days to 1.8 days, the direct medical costs for normal infants in 1991 was estimated at $479; this amount was subtracted from the mean charges for preterm infants to arrive at the estimate of the preterm LBW infants.

All 1991 charge data have been adjusted for 1992 using the overall medical care inflation rate of 6.6%.[17]

### Sensitivity Testing

To evaluate the limitations of these data, we analyzed the sensitivity of the results to the assumptions. First, the assumption of independence among the pregnancy complications was varied to look at the effect of double counting the cost of events occurring in individual patients. Second, the cost input data were varied 50% on either side of the 1991 mean charges for the various labor, delivery, and newborn complications included in the model in order to account for cost outliers that shift the means significantly one direction or another. In addition, the effect of discounting the hospital charges to align them with the payments for the complications was explored.

### RESULTS

The excess risk of pregnancy complications in women with BV was estimated from the published studies summarized in Table 1. The increased risk of preterm labor among women with BV in published studies has ranged from 2.0 to 2.6, while the risk for preterm delivery has ranged from 2.0 to 6.9 (Table 1). Other complications including preterm PROM, amniotic-fluid infection, and postpartum endometritis occurred 2–7 times more frequently among women with BV compared with women without this syndrome (Table 1).

The population attributable risks, which were estimated from the studies using the decision rules noted in Subjects and Methods, are summarized in Table 2.

Based on the U.S. population projections suggesting that 3.9 million pregnant women would have live births in 1993, we estimated the number of patients with pregnancy complications related to BV and the costs of those complications (Table 3). For these estimates, the assumption was made that the excess risks of pregnancy complications reported for women with BV are attributable to BV.

We also assumed in this base-case analysis that each of the perinatal complications occurs independently, resulting in an estimate of slightly fewer than 300,000 pregnancy complications attributable to BV; the costs of these complications are estimated to be $982 million for 1993. If, in fact, each pregnancy complication occurs independently, we can conclude that about 50% of the 585,000 pregnant women with BV have been affected by these perinatal complications. This assumption may re-

TABLE 3. Economic model for BV in pregnant women

| Complication | No. of patients with BV-associated complications | Cost per complication case[a] | Total costs (in millions) per complication |
|---|---|---|---|
| Preterm labor | 89,700 | $ 2,683 | $240.7 |
| Preterm delivery | 93,600 | $ 1,840 | $172.2 |
| LBW infants | 12,255 | $20,581 | $252.2 |
| Preterm PROM | 14,820 | $ 2,356 | $ 34.9 |
| Amniotic-fluid infection | 35,100 | $ 2,013 | $ 70.7 |
| Endometritis | 64,350 | $ 3,284 | $211.3 |
| Total costs | | | $982.0 |

[a]1991 charges adjusted for the rate of medical care inflation in 1992.

sult in some excess patient counts and costs being attributed to complications of BV since some patients will have more than one complication, costing collectively less than the sum of the excess cost for each complication. For example, a delivery after PROM may also involve an amniotic-fluid infection. We have chosen to deal with the effects of the independence assumption through sensitivity analysis.

Over 40% of the total direct costs estimated to be associated with BV in pregnant women involved the costs of preterm delivery and the attendant costs of intensive care for preterm LBW infants. Nearly a quarter of the costs were related to preterm labor (24.5%), while the remaining third of the costs were associated with preterm PROM, amniotic-fluid infection, and postpartum endometritis.

### Projections Through 2000

Assuming a constant rate of overall medical care inflation equal to the low level in 1993 (5.4%) and a constant BV incidence rate of 15% and retaining all other assumptions from the original model, we project that the direct costs of BV in pregnant women will reach $1.4 billion by the year 2000, as shown in Figure 1. If the incidence rate of BV-associated complications in pregnant women decreases 1% per year over the 8-year period as a result of changes in the population or effective screening and treatment, direct costs of about $1.3 billion are projected. If a more substantial decrease (10%) occurs in the rate of BV-associated complications in pregnant women each year, the direct costs could drop to approximately $679 million.

### Sensitivity Testing

Table 4 presents the results of the sensitivity analysis. In the column "1991 Cost Inputs," we have presented the base-case analysis. The next 2 columns present the alternative analyses, looking at extreme estimates. A key source of uncertainty in the assessment of the cost of BV in pregnant women is the extent to which the perinatal complications presented in Table 4 occur concomitantly within the same patient. For example, the epidemiology of these complications suggests that preterm LBWs are accompanied by preterm labor or preterm PROM.[18,19] There is further evidence that two-thirds of preterm deliveries are caused by preterm labor or preterm PROM.[20,21] We have used these probabilities for the likelihood of double counting in this model and assigned the lowest possible costs to each combination of perinatal complications to produce the outer range (minus 50%) for the analysis. The cost input data were also varied 50% on either side of the 1991 mean charges, adjusted for 1992 medical care inflation, to account for cost outliers that may have shifted the means for each pregnancy complication significantly one direction or another. That analysis suggests that the direct costs of BV-associated complications of pregnancy varied between $491 million and $1.5 billion in 1992 inflation-adjusted dollars.

In order to evaluate the limitations of using charge data rather than payment data for direct costs, we assumed that the difference between hospital charges and payments ranges from 5 to 10%. We reduced the 1992 inflation-adjusted charge data for each complication by 5% and 10% to reflect the direct costs of BV-associated complications if actual payment data had been used instead of charges; these cost figures are presented in the last 2 columns of Table 4. With all incidence and attributable risk estimates held constant, the estimated direct costs based on payment data ranged from $884 to $933

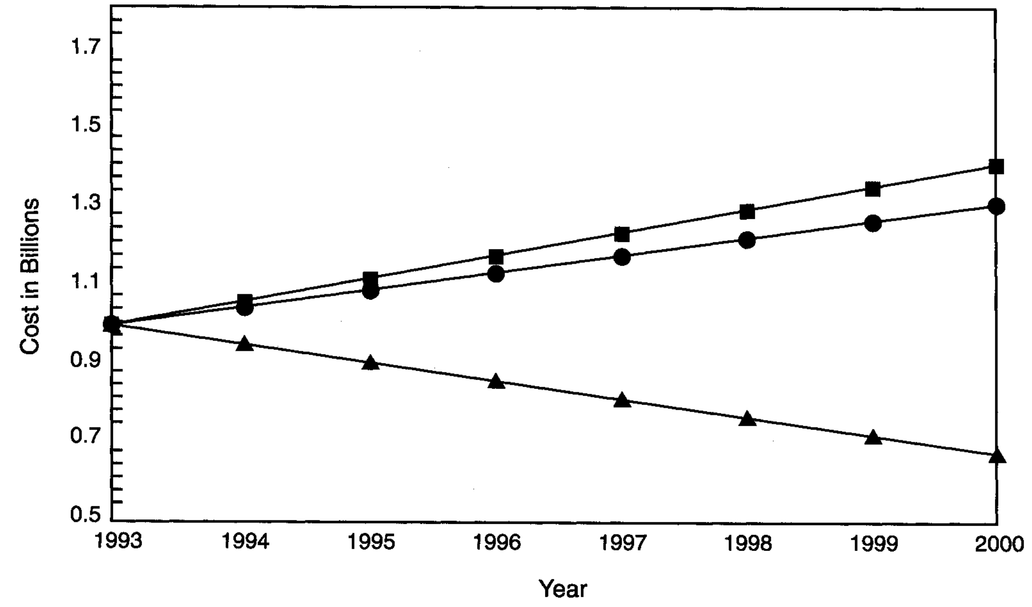

Fig. 1. Projected annual direct costs of BV-associated pregnancy complications based on predictions of the inflation rate and incidence changes. Squares = no change in incidence and constant low rate of inflation (5.4%); circles = 1% decrease in incidence per year and constant low rate of inflation; triangles = 10% decrease in incidence per year and constant low rate of inflation.

TABLE 4. Sensitivity analysis: Projected costs of BV-associated pregnancy complications assuming different cost inputs

| Complication | 1991 cost inputs[a] | Cost − 50% | Cost + 50% | Cost − 5% | Cost − 10% |
|---|---|---|---|---|---|
| Preterm labor | $ 2,683 | $ 1,342 | $ 4,025 | $ 2,549 | $ 2,415 |
| Preterm delivery | $ 1,840 | $ 920 | $ 2,760 | $ 1,748 | $ 1,656 |
| LBW infants | $20,581 | $10,291 | $30,872 | $19,552 | $18,523 |
| Preterm PROM | $ 2,356 | $ 1,178 | $ 3,534 | $ 2,238 | $ 2,120 |
| Amniotic-fluid infection | $ 2,013 | $ 1,007 | $ 3,020 | $ 1,912 | $ 1,812 |
| Epdometritis | $ 3,284 | $ 1,642 | $ 4,926 | $ 3,120 | $ 2,956 |
| Total costs[b] | $982 million | $491 million | $1,473 million | $933 million | $884 million |

[a]1991 charges adjusted for the rate of medical care inflation in 1992.
[b]Uses the most conservative assumptions from the analysis.

million for the BV-associated complications of pregnancy. Thus, the use of charge data may inflate the total cost estimate by $50 million to $100 million due to costs unrelated to actual patient care.

### DISCUSSION

Our analysis indicates that the direct economic cost of BV infections in pregnant women in the United States is nearly $1.0 billion annually. Although no studies are available for comparisons, the estimate we have computed is conservative for several reasons. First, we used the figures representing the lower range of incidence for BV detected at various stages of gestation in the pregnant population. Second, we have considered numerous confounding variables, i.e., socioeconomic factors, black race, young maternal age, other vaginal infections, and previous pregnancy performance, present among the cases of BV with complicated pregnancy outcomes and have assigned a population attributable risk to BV for the various pregnancy complications. Third, the costs associated with postsurgical infection following pregnancy termination have not been included since we have focused only on pregnancies resulting in live births. Finally, no psychosocial effects such as patient embarrassment, preoccupation with BV, or perceptions of the effect of BV on sexual activity were included. Such costs are known to exact a toll from patients and their families.

The reliability of the conclusions in this report clearly depend on the accuracy of the estimates. There are established epidemiologic criteria for determining whether a risk factor is causal for a given outcome. One criterion is finding the association consistently in different studies conducted in different populations over time. Another is the magnitude of the relative risk showing that the exposure occurs before the outcome. A further criterion is biologic plausibility, showing a biologic gradient in which increased exposure is associated with increased risk.

The series of studies reviewed for the incidence and attributable risks of pregnancy complications related to BV included women from different ethnic and socioeconomic groups and consistently reported an increased risk for preterm delivery or LBW among women with BV. In several of the cohort studies,[4–6] the assessment of BV was performed in the first or second trimester of pregnancy, documenting that BV was diagnosed prior to the onset of preterm labor. Likewise, the studies consistently show the relationship between BV and amniotic-fluid infection and postpartum endometritis. The association between upper-genital-tract infection and BV supports the biologic plausibility of the association between the syndrome and preterm birth because one mechanism by which BV causes preterm birth is thought to be by causing an upper-genital-tract infection.

BV is a syndrome caused by replacement of the vaginal lactobacilli by *Gardnerella vaginalis,* obligately anaerobic bacteria including *Prevotella bivia,* black-pigmented *Prevotella* and *Porphyromonas* species, *Bacteroides,* and genital mycoplasmas. The specific organisms associated with BV that are responsible for preterm delivery are unclear, although the anaerobic gram-negative rods are the group of microorganisms most consistently linked to preterm birth.[1,2] However, screening for vaginal anaerobes by culture is technically difficult and very expensive. Therefore, the use of the Gram-stained vaginal smear as a surrogate for the culture of anaerobic gram-negative rods is cost effective in identifying most women with high concentrations of vaginal anaerobes.[22]

The impact of variation from the projected cost data was investigated through the sensitivity analyses. Varying the cost input data by as much as 50% introduces substantial differences to our direct cost estimates; however, even at the lower extreme of minus 50%, the direct costs for BV-associated complications of pregnancy are estimated at nearly $500 million. As has been common practice in the literature examining the costs of providing hospital services, our study uses patient bills (charges) as a proxy for cost instead of measuring the costs directly. The use of charges introduces bias to the report, since hospital charge data include compensation for debts, replacement of equipment and facilities, free services, community programs, and disallowed (for reimbursement) costs, which may make the charges substantially more than the costs. In addition, the charges are adjusted to get a desired rate structure usually based on community norms. The potential impact of the bias due to overstating hospital costs on these results, which was also investigated with the sensitivity analyses, may represent $50–$100 million of the direct-cost estimates.

With the known morbidity caused by BV infections, these economic cost results underscore the need for effective BV treatment programs. The costs of BV in pregnant women are due to the consequences of untreated, uncomplicated infections that could have possibly been prevented. Even without increases in the incidence of BV, with no treatment effort the cost of BV in pregnant women will rise because of medical inflation. By 2000, the total cost of BV in pregnant women will reach $1.4 billion if the annual medical care inflation rate averages a low 5.4% from 1993 through 1999 and the incidence of BV remains constant during this period.

If the treatment of BV in pregnant women can be shown to reduce the incidence of these complications, then investments in treatment offer clear benefits. Routine screening of the nearly 4 million pregnant women in the United States each year could be easily accomplished by the Gram-stained vaginal smear.[23] At an estimated cost of $10 per smear, the screening of all pregnant women would cost $40 million annually. If 15% of the pregnant women have BV, then 600,000 women would be treated for this condition. With the costs of drug treatment ranging from $8 to $28 per woman, depending on the dosage and route of administration, the total drug treatment costs for all women with BV in the United States would range from

$4.8 to $16.8 million annually. Thus, screening all pregnant women in the United States for BV and subsequently treating those found to have the condition could be accomplished for $45–$57 million annually. The efficacy rate of oral metronidazole and clindamycin for the treatment of BV in pregnancy is 76–92%.[24,25] If the effectiveness rate assumption for these treatments is 65% and effective treatment decreases the frequency of preterm delivery attributable to BV by 50%,[24] then a conservative estimate of potential cost savings, after accounting for the costs of screening and treatment, would be $150 million annually.

The mounting body of evidence linking BV to pregnancy complications worldwide, combined with the excess financial and emotional costs associated with prematurity, suggest several research areas that deserve attention. First, the optimal time for screening and treatment has not been defined, although at least 2 studies suggest that early screening in the first or second trimester may be more predictive of preterm delivery than later screening.[4,5] This finding should be verified in a systematic way in other populations. Second, economic parameters need to be applied to the results of recently completed randomized treatment trials demonstrating the effect of drug therapy for BV during pregnancy in the prevention of pregnancy complications.[24,26] If these regimens are shown to decrease the incidence of pregnancy complications associated with BV, it will be possible to show the cost savings accruing to the health care payers.

Physicians are urged to learn to recognize the clinical signs of BV (milky vaginal discharge, a vaginal pH >4.5, and the release of an amine odor after the addition of 10% potassium hydroxide to the vaginal fluid), to screen selected patients, and to follow recommended treatment guidelines. Ultimately, it is hoped that the appropriate treatment of BV in pregnant women will be a cost-effective approach to reducing the incidence of preterm labor and the delivery of LBW infants.

### REFERENCES

1. Martius J, Krohn MA, Hillier SL, Stamm WE, Holmes KK, Eschenbach DA: Relationships of vaginal *Lactobacillus* species, cervical *Chlamydia trachomatis* and bacterial vaginosis to preterm birth. Obstet Gynecol 71:89–95, 1988.
2. Holst E, Goffeng AR, Andersch B: Bacterial vaginosis and vaginal microorganisms in idiopathic premature labor and association with pregnancy outcome. J Clin Microbiol 32:176–186, 1994.
3. Gravett MG, Nelson HP, DeRouen T, Critchlow C, Eschenbach DA, Holmes KK: Independent associations of bacterial vaginosis and *Chlamydia trachomatis* infection with adverse pregnancy outcome. JAMA 256:1899–1903, 1986.
4. Kurki T, Sivonen A, Renkonen O, Savia E, Ylikorkala O: Bacterial vaginosis in early pregnancy and pregnancy outcome. Obstet Gynecol 80:173–177, 1992.
5. Riduan JM, Hillier SL, Utomo B, Wiknjosastro G, Linnan M, Kandun N: Bacterial vaginosis and prematurity in Indonesia: Association in early and late pregnancy. Am J Obstet Gynecol 169:175–178, 1993.
6. Hay PE, Lamont RF, Taylor-Robinson D, Morgan DJ, Ison C, Pearson J: Abnormal bacterial colonization of the genital tract and subsequent preterm delivery and late miscarriage. Br Med J 308:295–298, 1994.
7. Hillier SL, Martius J, Krohn M, Kiviat N, Holmes KK, Eschenbach DA: A case-control study of chorioamnionic infection and histologic chorioamnionitis in prematurity. N Engl J Med 319:972–978, 1988.
8. Silver HM, Sperling RS, St. Clair PJ, Gibbs RS: Evidence relating bacterial vaginosis to intraamniotic infection. Am J Obstet Gynecol 161:808–812, 1989.
9. Watts DH, Krohn MA, Hillier SL, Eschenbach DA: Bacterial vaginosis as a risk factor for post-cesarean endometritis. Obstet Gynecol 75:52–58, 1990.
10. U.S. Bureau of the Census: Current Population Reports, Series P-25, No. 1018. Projections of the Population of the United States by Age, Sex, and Race: 1988 to 2080 by Gregory Spencer. Washington, D.C.: U.S. Government Printing Office, 1989.
11. Eschenbach DA: Bacterial vaginosis: Emphasis on upper genital tract complications. Obstet Gynecol Clin North Am 16:593–610, 1989.
12. Germain M, Krohn MA, Hillier SL, Eschenbach DA: Genital flora in pregnancy and its association with intrauterine growth retardation. J Clin Microbiol 32:2162–2168, 1994.
13. Gravett MG, Hummel D, Eschenbach DA, Holmes KK: Preterm labor associated with subclinical amniotic fluid infection and with bacterial vaginosis. Obstet Gynecol 67:229–237, 1986.
14. McGregor JA, French JI, Richter R, et al.: Antenatal microbiologic and maternal risk factors associated with prematurity. Am J Obstet Gynecol 163:1465–1473, 1990.
15. Committee on Technical Bulletins: Premature rupture of membranes. ACOG Tech Bull 115:1–5, 1988.
16. Fuchs VR, Perreault L: Expenditures for reproduction-related health care. JAMA 255:76–81, 1986.
17. Anon.: Prescription drug consumer price inflation falls to 15-year low of 5.2%. Health News Daily. Chevy Chase, MD: F-D-C Reports, Inc., January 19, 1993.
18. Berkowitz GS, Papiernik E: Epidemiology of preterm birth. Epidemiol Rev 15:414–443, 1993.

19. Meis PJ, Ernest JM, Moore ML: Causes of low birth weight births in public and private patients. Am J Obstet Gynecol 156:1165–1168, 1987.
20. Savitz DA, Blackmore CA, Thorp JM: Epidemiologic characteristics of preterm delivery: Etiologic heterogeneity. Am J Obstet Gynecol 164:467–471, 1991.
21. Main DM, Richardson D, Gabbe SG, Strong S, Weller SC: Prospective evaluation of a risk scoring system for predicting preterm delivery in black inner city women. Obstet Gynecol 69:61–66, 1987.
22. Hillier SL, Krohn MA, Rabe LK, Klebanoff SJ, Eschenbach DA: The normal vaginal flora, $H_2O_2$-producing lactobacilli, and bacterial vaginosis in pregnant women. Clin Infect Dis 16:S273–S281, 1993.
23. Nugent RP, Krohn MA, Hillier SL: Reliability of diagnosing bacterial vaginosis is improved by a standardized method of Gram stain interpretation. J Clin Microbiol 29:297–301, 1991.
24. McGregor JA, French JI, Parker R, et al.: Prevention of premature birth by screening and treatment for common genital tract infections: Results of a prospective controlled evaluation. Am J Obstet Gynecol 173:157–167, 1995.
25. McDonald HM, O'Loughlin JA, Vigneswaran R, Jolley PT, McDonald PJ: Bacterial vaginosis in pregnancy and efficacy of short-course oral metronidazole treatment: A randomized controlled trial. Obstet Gynecol 84:343–348, 1994.
26. Morales WJ, Schorr S, Albritton J: Effect of metronidazole in patients with preterm birth in preceding pregnancy and bacterial vaginosis: A placebo-controlled, double-blind study. Am J Obstet Gynecol 171:345–349, 1994.
